# Depression in Heart Failure with Reduced Ejection Fraction, an Undervalued Comorbidity: An Up-To-Date Review

**DOI:** 10.3390/medicina59050948

**Published:** 2023-05-15

**Authors:** Christian Basile, Antonio Luca Maria Parlati, Stefania Paolillo, Federica Marzano, Ermanno Nardi, Alfonsina Chirico, Davide Buonocore, Angela Colella, Sara Fontanarosa, Ciro Cotticelli, Anna Marchesi, Daniele Rodolico, Santo Dellegrottaglie, Paola Gargiulo, Maria Prastaro, Pasquale Perrone-Filardi, Roberta Montisci

**Affiliations:** 1 Department of Advanced Biomedical Sciences, University of Naples “Federico II”, 80131 Naples, Italy; 2 Department of Psychiatry, University Vita-Salute San Raffaele, 20132 Milan, Italy; 3 Department of Cardiovascular and Pulmonary Sciences, Catholic University of the Sacred Heart, 00128 Rome, Italy; 4 Advanced Cardiovascular Imaging Unit, Clinica Villa dei Fiori, 80011 Acerra, Italy; 5 Clinical Cardiology, AOU Cagliari, Department of Medical Science and Public Health, University of Cagliari, 09124 Cagliari, Italy

**Keywords:** heart failure, quality of life, depression

## Abstract

*Introduction*: Depression is a common and severe comorbidity among individuals with heart failure (HF). Up to a third of all HF patients are depressed, and an even higher proportion have symptoms of depression. *Aim*: In this review, we evaluate the relationship between HF and depression, explain the pathophysiology and epidemiology of both diseases and their relationship, and highlight novel diagnostic and therapeutic options for HF patients with depression. *Materials and Methods*: This narrative review involved keyword searches of PubMed and Web of Science. Review search terms included [“Depression” OR “Depres*” OR “major depr*”] AND [“Heart Failure” OR “HF” OR “HFrEF” OR “HFmrEF” OR “HFpEF” OR “HFimpEF”] in all fields. Studies included in the review met the following criteria: (A) published in a peer-reviewed journal; (B) described the impact of depression on HF and vice versa; and (C) were opinion papers, guidelines, case studies, descriptive studies, randomized control trials, prospective studies, retrospective studies, narrative reviews, and systematic reviews. *Results*: Depression is an emergent HF risk factor and strongly relates with worse clinical outcomes. HF and depression share multiple pathways, including platelet dis-reactivity, neuroendocrine malfunction, inappropriate inflammation, tachi-arrhythmias, and frailty in the social and community setting. Existing HF guidelines urge evaluation of depression in all HF patients, and numerous screening tools are available. Depression is ultimately diagnosed based on DSM-5 criteria. There are both non-pharmaceutical and pharmaceutical treatments for depression. Regarding depressed symptoms, non-pharmaceutical treatments, such as cognitive-behavioral therapy and physical exercise, have shown therapeutic results, under medical supervision and with an effort level adapted to the patient’s physical resources, together with optimal HF treatment. In randomized clinical studies, selective serotonin reuptake inhibitors, the backbone of antidepressant treatment, did not demonstrate advantage over the placebo in patients with HF. New antidepressant medications are currently being studied and could provide a chance to enhance management, treatment, and control of depression in patients with HF. *Conclusions*: Despite the substantial link between depression and HF, their combination is underdiagnosed and undertreated. Considering the hopeful yet unclear findings of antidepressant trials, further research is required to identify people who may benefit from antidepressant medication. The goal of future research should be a complete approach to the care of these patients, who are anticipated to become a significant medical burden in the future.

## 1. Introduction

Depression is a mental disease characterized by a broad range of fluctuating symptoms and an elevated risk of mortality and morbidity [1]. The idea that emotions have a major effect on the heart stretches back to the earliest days of medicine [2]. Malzberg found a higher death rate related to depression in 1937, mostly owing to cardiovascular reasons [3]. In the 1990s, initial studies on the relationship between depression and heart failure (HF) became available, finding that around 30% of HF patients had depression symptoms [4]. Current guidelines from both the European Society of Cardiology (ESC) [5] and the American College of Cardiology/American Heart Association/Heart Failure Society of America (ACC/AHA/HFSA) [6] recommend screening for and treating depression in HF patients. However, there are numerous obstacles in the diagnosis (relapsing and remitting course, divergency in symptoms and their overlap) and management of patients with comorbid HF and depression [7].

In this review, we investigate the relationship between HF and depression, explain the physiology and epidemiology, and highlight novel diagnostic and therapeutic options for patients with both depression and HF. Since the bulk of depression research has been performed with patients with HF with reduced ejection fraction (HFrEF), this review focuses mostly on this cohort.

## 2. Materials and Methods

### 2.1. Study Design

The aim of the present narrative review is to evaluate the latest available evidence on the impact of depression on cardiovascular diseases and more specifically HF with reduced ejection fraction.

### 2.2. Search Strategy

This narrative review involved keyword searches of PubMed and Web of Science. Review search terms included [“Depression” OR “Depres*” OR “major depr*”] AND [“Heart Failure” OR “HF” OR “HFrEF” OR “HFmrEF” OR “HFpEF” OR “HFimpEF”] in all fields.

The databases were searched without any restrictions from inception to 1 February 2023. Two authors (C.B. and A.L.M.P.) separately examined the titles and abstracts of all obtained publications to exclude clearly unrelated research. No language restrictions were applied. Study abstracts in languages other than English and Italian were translated by native language speakers available at the University of Naples “Federico II” campus to evaluate the suitability of the study. References of the provided articles were also examined to discover any additional relevant studies.

### 2.3. Study Selection

**Figure 1:**
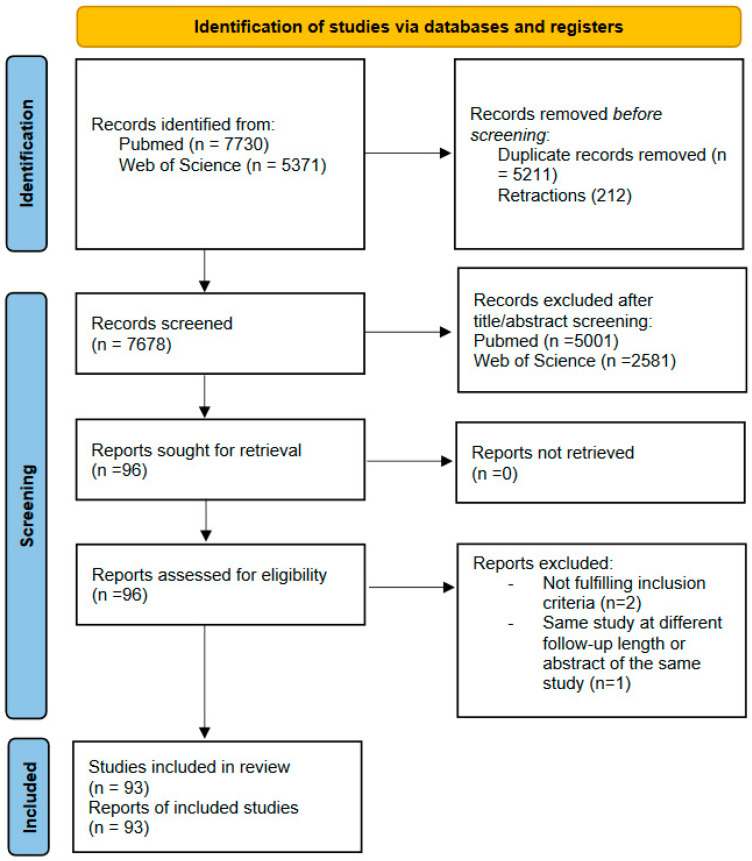
PRISMA 2020 flow diagram for new systematic reviews, which included searches of databases and registers only.

Studies included in the review met all the following criteria: (A) published in a peer-reviewed journal; (B) described the impact of depression on HF and vice versa; and (C) were opinion papers, guidelines, case studies, descriptive studies, randomized control trials, prospective studies, retrospective studies, narrative reviews, and systematic reviews. The search process is shown in Figure 1.

## 3. Results

### 3.1. Epidemiology

The global incidence of depression is over three times that of HF [8]; hence, the predicted prevalence of comorbid depression and HF will expand considerably over the next several years, as will its economic cost [9]. When combined, HF and depression double healthcare expenses compared to each condition taken individually [10]. Numerous risk factors, including female gender [11], age [12], and past depressive events, increase the likelihood of depression. Additionally, depression appears to raise HF risk, and HF appears to increase the risk of depression [13]. These reciprocal connections suggest a complicated interaction between the two diseases. Despite the difficulty in detecting depression in HF patients, it is essential to identify depression since it remains linked to dramatically augmented morbidity and death in patients with HF [14].

### 3.2. Depression in Heart Failure

Numerous studies indicate that HF relates to an increased risk of developing depression. Two considerable meta-analyses [15,16] and two longitudinal studies [17,18] determined that the likely global prevalence of depression in patients with HF is between 20 and 30 percent (Table 1).

Intriguingly, this prevalence is comparable in HF across a wide range of ejection fraction patients, as well as across various HF etiologies, with women and the elderly at a greater risk for depression both in the HF and in the general population, thus associating HF with an increased risk of depressive symptoms and depression. In addition, the severity of HF symptoms, ischemic etiology, or previous hospitalization are risk factors for depression in patients with HF [15,16,17,18].

### 3.3. Influence on Cardiovascular Outcomes of Concomitant HF and Depression

Depression is associated with the onset and advancement of many cardiovascular disorders and especially of HF. In a prospective observational study, even when corrected for other cardiovascular risk factors, depression was related to an increased risk of 18% in the development of HF over the 7-year follow-up [19].

In separate research among nearly 80,000 healthy veterans without cardiac pathology, a severe depression diagnosis was associated with a 21% higher risk of developing HF in the subsequent 5.8-year follow-up [20]. Depression is also a poor prognostic indicator among people with a diagnosis of HF [21]. Prospective research has associated higher depressed symptoms and depressive disorders to recurrent hospitalizations, cardiac events, and death in this population [22,23].

The combination of HF and depression is related to an increased burden for both disease and increased mortality. In two meta-analyses by Rutledge et al. [15] and Sokoreli et al. [16], the composite outcome of cardiovascular events and all-cause mortality was shown to be higher in patients with both depression and HF. Notably, the higher death risk in individuals with both depression and HF appears to be elevated in the short term as well as in the long term.

Equally, in the study conducted by Jiang et al. [18] depression was linked with higher 3-month (OR 2.5) and 1-year (OR 2.23) mortality as well as higher rehospitalization for heart failure rates at 3 months (OR 1.90) and at 1 year (OR 3.07) (all *p* < 0.05), with these augmented risks being independent of significant moderators such as NYHA class, age, ejection fraction phenotype, or etiology of HF.

Other evidence has linked depression with negative outcomes following heart transplantation [24]. In addition, multiple case-control trials have demonstrated that HF is associated with a higher suicide risk, particularly during a fragile window after the HF diagnosis (6–24 months) [25], and that this risk is significantly affected by the burden of depressive symptoms [26]. Burden and category of the depressive symptomatology, gender, and age are just a few of the factors that could impact the effect of depression on HF patients [27,28], considering that the burden of depressive symptoms was linked with increased mortality in HF patients (HR 4.06 for severe depressive symptoms) [28]. Somatic symptoms, as opposed to cognitive symptoms, are more strongly linked with outcomes in HF patients with depression [29]. Depression and HF also influence quality of life (QOL). For instance, Dekker et al. demonstrated that improvement in depressive symptoms is associated with an improvement in QOL evaluated with the PHQ-9 questionnaire; improvement was significant after adjusting for possible confounders [30].

### 3.4. Mechanism of Comorbid Depression and HF

HF and depression share various pathways, such as alterations in platelet activation, inflammatory response, neuroendocrine system, heart rhythm, and adherence to therapy [31].

Depression is strongly associated with coronary heart disease (HD), which is the leading risk factor for the development of HF [32]. In HD patients, depression is related with increased platelet activation [33]. In addition, sertraline seems to reduce platelet hyperactivation in patients with depression and ischemic HF, regardless of concomitant antiplatelet therapy [34]. Larger investigations that controlled for confounding variables failed to show differences in platelet activation related to the depressive status of individuals with HD [35].

Autonomic nervous system changes likewise play a role in the associations between depressive symptoms and outcomes. HF is often associated with a dysregulation of the autonomic nervous system, described as a mismatch in the sympathetic/parasympathetic activity (hyperactivity of the sympathetic—hypoactivity of the parasympathetic). This imbalance has been associated with mortality in HF, as well as heart remodeling and arrhythmias [36].

An association exists between negative psychological states and autonomic disfunction. Depression, for instance, has been linked to a lower variability in heart rate, an indicator of aberrant autonomic function, in people regardless of cardiac disease status [37]. These decreases in heart rate variability are more pronounced as the burden of depressive symptoms increases, indicating that patients with depressive disorder may be at a heightened risk for this dysfunction [38]. Through the stimulation of distinct detrimental responses, the hypothalamic–pituitary axis is a potential mechanism at the crossroads of HF and depression. Depression is linked with elevated levels of noradrenaline–adrenaline and may therefore negatively impact HF patients [23,39,40]. Moreover, in HF patients with depressive symptoms immune cells are more sensitive to adrenergic stimuli than immune cells of healthy individuals [41]. Even after controlling for HF severity, individuals with more severe depression symptoms had a greater resting heart rate. Multiple studies have linked depression and HF with an increased risk of arrhythmias. Depression was related with a higher risk for sudden cardiac mortality [42], ventricular tachi-arrhythmias [43], and atrial fibrillation relapse [44]. The higher mortality among patients with both HF and depression could therefore be explained by the increased risk of ventricular tachi-arrhythmias, even if this pro-arrhythmic state’s underlying mechanism is poorly comprehended.

Additionally, endothelial disfunction may play a crucial role. An appropriate endothelial function is essential to guarantee proper peripherical and central perfusion and to lower cardiac strain. Endothelial dysfunction in individuals with HF is associated with higher rates of hospitalizations for HF and all-cause death [45,46]. Depression is linked to endothelial dysfunction in both HF and non-HF individuals [47]. Depressive symptoms relate to a lower L-arginine/ADMA ratio in patients with HF, suggesting decreased nitric oxide availability and endothelial dysfunction [48]. The burden of depressive symptoms is also related to abnormalities in flow-mediated distention, an indicator of endothelial function in HF patients [49].

The deregulation of inflammatory pathways is a significant possible mechanism linking HF and depression [50]. In fact, it seems that elevated levels of inflammatory markers are a risk factor for both these diseases. Numerous studies have demonstrated a correlation between various inflammatory markers and cardiovascular outcomes in patients with comorbid depression and HF [51,52]. Nonetheless, evidence regarding inflammation’s role is drawn from minor non-randomized trials; therefore, bigger confirmation studies are necessary.

It has been reported that social factors are a risk for either HF or depression. Social isolation relates to sadness and poor outcomes in people with HF [53]. The likelihood of depression and HF co-occurring may be affected by risky behaviors [54] and absence of adherence to therapy. In depressed patients, smoking and inactivity are potential risk factors for HD [55]. HF patients treated with antidepressants do not receive guideline-driven medical treatment due to potential interference and are more likely to not adhere to their medications [56,57]. Comorbid depression decreases lifestyle advice and HF rehabilitation adherence [58]. Additionally, depression and HF are autonomously associated with cognitive impairment, leading to lower patient compliance [59,60]. Inconsistent data indicate that cognitive impairment is either a mediator or confounder of the connection linking depression and HF. Moreover, current evidence suggests that brain involvement and cognitive deficits are different in patients with HF and depression [61].

### 3.5. Depression and Heart Disease in Patients with No Previous Cardiac Diseases

Depression in asymptomatic and healthy people seems to be a powerful and independent predictor of HD.

As shown by Nicholson et al. [62] in a meta-analysis of prospective studies, depression increased the relative risk for HD by 1.64, which was midway between more conventional risk factors such as passive (1.25) and active (2.5) smoking.

In the Amsterdam longitudinal aging study [63], a dose–response model seems to better fit the effect of depression on HD, with direct correlation between the years of depression and magnitude of depressive symptoms and the incidence of HD with an adjusted relative risk of cardiovascular mortality of 3.8 for patients diagnosed with depression.

In the Whitehall II study [64], age-adjusted and sex-adjusted HR for all-cause mortality were 1.67 for participants with only HD, 2.10 for those with only depression, and 4.99 for patients with both HD and depression when compared to patients without depression and without HD. Therefore, the surplus for the all-cause mortality HR is reasonably attributable to the interaction between depression and HD, with clear evidence of an additive interaction.

There is therefore evidence that depression is related to an increased risk of all-cause and cardiovascular mortality, and that this risk, albeit more pronounced in patients with HD, is still relevant in patients with no previous cardiac disease.

### 3.6. Diagnosis

Present ESC and ACC/AHA/FSHA HF recommendations urge raising knowledge of depression and depressive symptoms in patients with HF; hence, individuals with HF should be frequently tested for depression. While HF diagnosis is sustained by several findings (signs, symptoms, biomarkers, and imaging), the diagnosis of depression relies on questionnaires and interviews [65].

In the setting of HF, it remains difficult to distinguish between a natural response to chronic disease and depression, particularly early after hospitalization for HF [66]. Troubles in attributing symptoms to either depression or HF could confound the diagnosis due to a possible intersection in manifestation from both diseases. Different screening questionnaires for clinically significant depression symptoms have been validated (Table 2).

The Patient Health Questionnaire-2 (PHQ-2) is a straightforward screening instrument for depressive symptoms that measures them on a 6-point scale. A PHQ-2 score of at least 3 seems to be an excellent screening tool, indicating the necessity of a more specialized evaluation, such as the Patient Health Questionnaire-9 (PHQ-9), that evaluates many depressive domains in a more comprehensive manner on a wider 27-point scale. The total PHQ-9 result could be used to rate depression symptom severity; hence, it can be used to identify individuals with severe symptoms requiring immediate treatment [67].

For instance, this is what the AHA advises with its two-step depression screening system for patients with heart disease [68]. This approach has been shown to be quite specific (91%), but only modestly sensitive (52%). Even though this approach has not been explicitly evaluated in HF individuals, the PHQ-9 has an acceptable accuracy for detecting significant depressive symptoms in this population [69].

Notably, the existence of depressed symptoms is not a depression diagnosis. Questionnaires frequently assess symptoms that are not taken into account during the clinical interview; as a result, they may over-diagnose depression.

The most precise way to diagnose depression is the clinical interview. The Diagnostic and Statistical Manual of Mental Disorders, Fifth Edition (DSM-5) defines depression as the presence for at least two weeks of major depressive symptoms combined with at least four minor symptoms (appetite, weight or sleep behavior changes, fatigue, guilt or worthlessness, psychomotor or concentration impairments, or suicidal ideation) [70]. Multiple studies have shown the varied courses of depressive symptoms in people with HF [71]. After one year, over half of depressed individuals with HF show a reduction of depressive symptoms. Therefore, depression therapy for HF patients should be tailored and evaluated on the basis of cyclic risk–benefit analysis.

### 3.7. Treatment

The standard therapy for depression involves both pharmaceutical and non-pharmaceutical interventions. A limited number of randomized controlled studies have examined the impact of various therapies on depression symptoms. Physical exercise and cognitive-behavioral therapy (CBT) were linked to a substantial improvement in depressive symptoms in a network meta-analysis of 21 randomized HF studies [72]. Although antidepressants lessened symptoms, their impact was not statistically significant.

#### 3.7.1. Non-Pharmacological Therapy

CBT, physical exercise, and palliative care are the most important non-drug therapies in patients with depression.

Exercise treatment may improve symptoms and outcomes in patients with both HF and depression [73]. The “Heart Failure: A Controlled Trial Investigating the Outcomes of Exercise Training” (HF-ACTION) trial randomized 2331 patients with HFrEF to exercise training or conventional therapy. Even in individuals with clinically severe depressed symptoms, exercise training at 3 and 12 months substantially decreased the severity of depressive symptoms [74]. Notably, the higher decrease was related to increased exercise duration, with the benefits reaching a plateau after 90 min per week. A randomized trial, albeit small, suggests that in HF patients even light exercise may be useful for depressive symptoms [75]. However, caregivers may have difficulty convincing HF patients with depression to exercise.

CBT is a psychiatric intervention that aims to modify abnormal cognitive and behavioral processes. A meta-analysis of one observational trial and five randomized trials revealed only a small recovery from symptoms of depression and increased QOL when HF patients with depression were treated with CBT [76]. In the biggest randomized study to date, Freedland and colleagues determined that CBT was superior to standard treatment in lowering depression manifestations over a 12-month period [77]. Furthermore, CBT was linked with lower rehospitalizations (RR 0.47, *p* = 0.001). Considering the inadequate and contradictory findings on the effectiveness of CBT, new trials with bigger sample sizes and a more extensive follow-up period are required.

In the Palliative Care in Heart Failure (PAL-HF) trial, a palliative therapy strategy decreased depressive symptoms in advanced HF patients compared to standard care [78]. This method includes a physician, a palliative care nurse, and a psychiatrist, with the potential use of antidepressants, anxiolytics, and pain management based on the available treatment options.

#### 3.7.2. Pharmacological Therapy

Due to the increased risk of ventricular tachi-arrhythmia, severe hypotension, and acute coronary syndrome, tricyclic antidepressants, the first antidepressants to be licensed, are contraindicated in individuals with HF. Selective serotonin reuptake inhibitors (SSRIs) have a more favorable safety profile. However, many randomized trials comparing SSRIs to the placebo in addition to the HF standard of care in depressed individuals with HF showed no benefit (Table 3).

Following promising findings in depressed individuals after an acute coronary syndrome [79], sertraline was evaluated in depressed HF individuals. In the Sertraline Against Depression and Heart Disease in Chronic Heart Failure (SADHART-CHF) study [80], sertraline medication alleviated depression symptoms in HF patients, although the benefits did not vary from those of standard care (placebo and nurse-facilitated support). Death and HF hospitalization were comparable between therapy groups. However, these findings may have been affected by the population’s modest severity of depression symptoms and the low amount of sertraline administered.

Escitalopram was evaluated for use with HF patients after showing a somewhat beneficial effect in ischemic patients. In the Effects of Selective Serotonin Reuptake Inhibition on Morbidity, Mortality, and Mood in Depressed Heart Failure Patients (MOOD-HF) trial [81], escitalopram showed no improvement in depressive symptoms in HF patients with depression.

There are contradictory findings on the safety of antidepressants in HF patients. A recent meta-analysis revealed an augmented all-cause mortality among HF individuals treated with antidepressants, regardless of antidepressant class [82]. Based on this limited data, more research is required to rule out an adverse impact of antidepressants in HF.

#### 3.7.3. Integrated Care Models in the Management of Depression in Patients with Heart Disease

Collaborative care is a potential way to provide psychiatric therapy to depressive patients. This paradigm employs a non-physician care manager, under the supervision of a mental health specialist, to screen mental problems and manage novel drugs within the patient’s existing therapy. In primary care settings, where collaborative care initiatives have been most frequently implemented, depression and QOL have improved [83]. Collaborative care programs are equally effective for patients with depression and HD; one program that targeted both depression and cardiovascular risk factors led to improvements in a variety of factors including mental health, blood pressure, and LDL cholesterol [84]. The Patient-Centered Disease Treatment trial assessed the effectiveness of a multi-component collaborative care intervention for the management of symptoms of depression and HF [85]. The intervention showed no improvement in QOL compared to usual care.

#### 3.7.4. New Therapies

Approximately one third of individuals present with multiple drug-resistant depression. NMDA receptor antagonists, modern brain stimulation, and omega-3 fatty acid supplements are being researched as potential new antidepressant therapies with better effectiveness and safety profiles.

Traditionally, brain stimulation treatments, such as electroconvulsive therapy, were utilized to treat resistant depression with a prompt response in about 50% of the treated individuals [86]. Potential side effects and the necessity for hospitalization and monitoring have hampered the effectiveness of this therapy for HF individuals. Modern brain stimulation technology transcranial magnetic stimulation (TMS) has overcome these restraints and is an effective therapy for depression. Using an electromagnetic coil, TMS creates repeating electrical fields that influence neuronal circuits in the brain. TMS is a potential therapy for HF patients since it does not need anesthesia and has no side effects (such as seizures) [87]. It should be noted though that implanted devices such as pacemakers, loop recorders, and defibrillators are a significant contraindication for TMS therapy.

NMDA receptor antagonists, based on first finding that ketamine is able to reduce depressive symptoms, had promising outcomes in several small trials. Esketamine, the intranasal S-enantiomer of ketamine, has been recently authorized for the treatment of resistant depression in the United States [88]. Despite encouraging findings, the possible side effects such as hypertension or prolonged QTc interval remain the greatest worry about the safety of these medications, especially in patients with established HD or HF. Future studies are required to elucidate the antidepressant benefits and any side effects in HF patients.

Multiple investigations have shown that depressed HF patients have an overall reduction in omega-3 fatty acid storage. A sub-analysis of the SADHART-CHF research showed that low levels of omega-3 fatty acids were related to increased mortality in HF patients with depression [89]. Several minor studies have shown that antidepressants are more effective when combined with polyunsaturated fatty acids in depressed individuals. In the Omega-3 Supplementation for Co-Morbid Depression and Heart Failure Treatment (OCEAN) study, 108 HF patients with depression (35% with HFpEF and 65% with HFrEF) were randomized to 12 weeks of treatment with omega-3 or a placebo [90]. Omega-3 supplementation was linked to improvements in cognitive depression symptoms and the 6 min walk test.

## 4. Discussion

Depression has been proven to be a meaningful factor to consider in HF patients to better stratify prognosis in patients with HF and related comorbidities.

In recent years, research has been focused on this disease to understand the pathophysiological processes underlying the interconnections between HF and depression. The current HF guidelines do not recommend screening for depression in HF; however the current guidelines on cardiovascular disease prevention recommend the evaluation of mental health as a risk modifier. This, as latest research has shown, is particularly relevant in HF patients with depression. The bulk of evidence shown in our review clearly indicates the mortality and morbidity burden of depression in HF patients, raising awareness among clinicians regarding the use of easy-to-use screening tools to better asses depressive symptoms in those patients and to further evaluate them, redirecting those patients to valuable specialized clinicians, in cases of high likelihood of depression.

Considering the data reported in the current manuscript, we suggest screening HF patients for depression at least once after the first 6 months of HF, and to regularly screen depressed patients for cardiovascular implications based on their risk profile. Once the screening has suggested depression in the HF patient, we suggest an active interaction between the HF specialist and the psychiatrist to better manage the additive effect of these two pathologies, even if there is a lack of evidence of the utility and efficacy of a specific cardio-psychiatric team in this patient setting. It should also be considered that, as shown in [83,84,85], an integrated care model is crucial for those patients; however, not all health systems are capable of applying such an integrated model, often relying on caregivers and family for the external support needed to manage therapy compliance and accurate symptom detection.

Our review suffers from some limitations: being a narrative review, the protocol was not registered on PROSPERO; neither were quantitative analysis performed. Furthermore, previous research has almost solely focused on HFrEF, and little data on depression in HFpEF patients have been published; albeit, some research suggests that depression is more prevalent in patients with HFpEF [91]. Similarly, depression appears to increase the likelihood of worse outcomes for HFpEF individuals [92]. Nonetheless, the magnitude of this association’s influence is not fully established. Studies on exercise training and depressive symptoms in HFpEF patients have shown poor results [93], and no trials have been conducted on the use and efficacy of antidepressant medications for patients with HFpEF to date.

Lastly, considering the lack of robust, sufficiently powered RCT to evaluate hard outcomes (cardiovascular mortality, cardiovascular events, hospitalization for HF, and all-cause mortality) on intervention vs. placebo in patients with comorbid depression and HF, all the evidence shown regarding those hard outcomes should be taken with a grain of salt and be considered hypothesis-generating.

## 5. Conclusions

Despite the substantial link between depression and HF, their combination is underdiagnosed and undertreated. Although an HF comorbidity management programme could be effective for the diagnosis and treatment of concomitant depression, a plan with a multidisciplinary approach did not improve outcomes or symptoms.. Future research is required to investigate the mechanisms of depression in HFpEF and their therapy. Existing ESC and ACC/AHA/HFSA HF guidelines acknowledge the need for more effective medications to reduce morbidity and mortality in depressed HF patients.

Considering the hopeful yet unclear findings of antidepressant trials, further research is required to identify people who may benefit from antidepressant medication. The goal of future research should be a complete approach to the care of these patients, who are anticipated to become a significant medical burden in the future.

## Figures and Tables

**Table 1:** Prevalence of depression in heart failure.

Author	Year	Design	Sample Size	Prevalence
Jiang et al. [16]	2001	Longitudinal study	682	20%
Freedland et al. [15]	2003	Longitudinal study	375	35.3%
Rutledge et al. [13]	2006	Meta-analysis	6202	21.5%
Sokoreli et al. [14]	2016	Meta-analysis	80,627	29%

**Table 2:** Comparison of questionnaires for depression diagnosis. HD, coronary heart disease; DSM, Diagnostic and Statistical Manual of Mental Disorders.

Name	Elements	Time (Min)	Result	Pros	Cons
Patient Health Questionnaire-2 (PHQ-2)	2	<1	0–3	- Easy to use - Fast	- Self-reporting - Low sensitivity in patients with CHD
Patient Health Questionnaire-9 (PHQ-9)	9	5	0–3	- Diagnosis - Symptom severity assessment	- Self-reporting
Hospital Anxiety and Depression Scale (HADS)	14 (7 for depression and 7 for anxiety)	5	0–3	- Easy to use - Fast - Reliable - Focus on anhedonia	- No physical, cognitive, and suicidal symptoms evaluation - Not ideal for cardiac units
Beck Depression Inventory-II (BDI-II)	21	15	0–4	- Simple and clear - Physical - and cognitive symptoms evaluation - Symptom severity evaluation - High reliability	- Self-reporting
Hamilton Rating Scale for Depression (HAM-D)	17	15	0–1	- Short - Symptom - severity evaluation and response to treatment	- Reliability is controversial - Requires training - Lack of secondary symptom domains related to depression
Geriatric Depression Scale (GDS)	15 or 30	5	0–3	- Easy - Suitable for primary- - care-based depression screening	- No evaluation of somatic symptoms
Cardiac Depression Scale (CDS)	26	5	26–182	- Easy - Reliable - Specific for cardiac patients - Useful as a screening instrument	- Self-reporting

**Table 3:** Randomized controlled trials on depression in heart failure. BDI-II, Beck Depression Inventory-II; DHA, docosahexaenoic acid; EF, ejection fraction; EPA, eicosapentaenoic acid; HAM-D, Hamilton Rating Scale for Depression; HF, congestive heart failure; HR, hazard ratio; MADRS, Montgomery–Asberg Depression Rating Scale; PHQ-9, Patient Health Questionnaire-9.

Trial	Inclusion Criteria	Number of Patients	Drug	Mean Follow-Up	Primary Endpoints	Results
SADHART-CHF	- HF - EF ≤ 45% - New York Heart Association functional classification II–IV - Clinical interview	469	Sertraline vs. placebo	12 weeks	- Mean ± SE change in HAM-D - Composite cardiovascular status	No significant differences between groups (*p* = 0.78)
MOOD-HF	- HF - EF ≤45% - New York Heart Association functional classification II–IV - Clinical interview and PHQ-9	372	Escitalopram 10–20 mg/day vs. placebo	24 months	- All-cause mortality or hospitalization - Mean MADRS score variation	No significant between-group difference (*p* = 0.26)
OCEAN	- HF across the EF spectrum - New York Heart Association functional classification II–IV - Clinical interview + HAM-D ≥ 18	108	High EPA vs. placebo	12 weeks	- HAM-D and BDI-II	No significant between-group difference

## Data Availability

Not applicable.
